# Odontoblasts release exosomes to regulate the odontoblastic differentiation of dental pulp stem cells

**DOI:** 10.1186/s13287-023-03401-9

**Published:** 2023-07-08

**Authors:** Xinghong Luo, Weiqing Feng, Shijiang Huang, Shenghong Miao, Tao Jiang, Qian Lei, Jingyao Yin, Sheng Zhang, Xiaochun Bai, Chunbo Hao, Weizhong Li, Dandan Ma

**Affiliations:** 1 Department of Endodontics, Stomatological Hospital, School of Stomatology, Southern Medical University, No 366 Jiangnan Avenue South, Guangzhou, 510280 Guangdong China; 2 School of Stomatology, Southern Medical University, Guangzhou, China; 3 Department of Cell Biology, School of Basic Medical Science, Southern Medical University, Guangzhou, China; 4 Department of Stomatology, Nanfang Hospital, Southern Medical University, No.1838 North Guangzhou Avenue, Guangzhou, People’s Republic of China; 5 Department of Stomatology, Hainan General Hospital, Hainan Affiliated Hospital of Hainan Medical University, Haikou, Hainan China

**Keywords:** Exosomes, mTORC1, Odontoblasts, DPSCs, Odontoblastic differentiation

## Abstract

**Background:**

Dental pulp stem cells (DPSCs) play a crucial role in dentin-pulp complex regeneration. Further understanding of the mechanism by which DPSCs remain in a quiescent state could contribute to improvements in the dentin-pulp complex and dentinogenesis.

**Methods:**

TSC1 conditional knockout (DMP1-Cre+; TSC1^f/f^, hereafter CKO) mice were generated to increase the activity of mechanistic target of rapamycin complex 1 (mTORC1). H&E staining, immunofluorescence and micro-CT analysis were performed with these CKO mice and littermate controls. In vitro, exosomes were collected from the supernatants of MDPC23 cells with different levels of mTORC1 activity and then characterized by transmission electron microscopy and nanoparticle tracking analysis. DPSCs were cocultured with MDPC23 cells and MDPC23 cell-derived exosomes. Alizarin Red S staining, ALP staining, qRT‒PCR, western blotting analysis and micro-RNA sequencing were performed.

**Results:**

Our study showed that mTORC1 activation in odontoblasts resulted in thicker dentin and higher dentin volume/tooth volume of molars, and it increased the expression levels of the exosome markers CD63 and Alix. In vitro, when DPSCs were cocultured with MDPC23 cells, odontoblastic differentiation was inhibited. However, the inhibition of odontoblastic differentiation was reversed when DPSCs were cocultured with MDPC23 cells with mTORC1 overactivation. To further study the effects of mTORC1 on exosome release from odontoblasts, MDPC23 cells were treated with rapamycin or shRNA-TSC1 to inactivate or activate mTORC1, respectively. The results revealed that exosome release from odontoblasts was negatively correlated with mTORC1 activity. Moreover, exosomes derived from MDPC23 cells with active or inactive mTORC1 inhibited the odontoblastic differentiation of DPSCs at the same concentration. miRNA sequencing analysis of exosomes that were derived from shTSC1-transfected MDPC23 cells, rapamycin-treated MDPC23 cells or nontreated MDPC23 cells revealed that the majority of the miRNAs were similar among these groups. In addition, exosomes derived from odontoblasts inhibited the odontoblastic differentiation of DPSCs, and the inhibitory effect was positively correlated with exosome concentration.

**Conclusion:**

mTORC1 regulates exosome release from odontoblasts to inhibit the odontoblastic differentiation of DPSCs, but it does not alter exosomal contents. These findings might provide a new understanding of dental pulp complex regeneration.

**Supplementary Information:**

The online version contains supplementary material available at 10.1186/s13287-023-03401-9.

## Introduction

It is well known that most dental pulp stem cells (DPSCs), which are adult stem cells (ASCs), exist in a quiescent state under normal physiological conditions [1]. Once DPSCs are stimulated, they can differentiate into odontoblasts. The odontoblastic differentiation of DPSCs is regulated by a variety of signaling molecules, transcription factors and exosomes in the dental pulp microenvironment. Transforming growth factor (TGF-β), tumor necrosis factor-α (TNF-α) and lipopolysaccharide (LPS) have been reported to regulate the differentiation of DPSCs in the dental pulp microenvironment [2]. However, the regulatory mechanism by which DPSCs remain in a quiescent state remains unknown.

Mechanistic target of rapamycin (mTOR), which is a highly conserved Ser/Thr protein kinase, can form two complexes, mTOR complex 1 (mTORC1) and mTOR complex 2 (mTORC2) [3]. Tuberous sclerosis complex 1 (TSC1) normally downregulates mTORC1 [4, 5]. When cells are stimulated, PI3-K/Akt is activated and inhibits TSC1 activity; this protects Ras homolog enriched in brain (RHEB) from inhibition by TSC1, and then, mTORC1 is activated [6]. The expression level of phospho-S6 (pS6), observed by western blotting analysis, is often considered a marker of mTORC1 activity [7]. It is well known that autophagy is mainly regulated by mTORC1. When mTORC1 is activated by TSC1 inhibition, autophagy is greatly decreased [8]. mTORC1 has been reported to control the release of exosomes in order to regulate the response of liver cells to differences in the levels of external nutrients and growth factors [9]. It has also been reported that mTORC1 inhibition can cause the release of exosomes to promote stress-induced tumor adaptation [10]. However, the relationship between mTORC1 and exosomes in the odontoblastic differentiation of DPSCs has not been reported. mTORC1 is essential for the osteo/odontoblastic differentiation of dental stem cells [11–13]. In our recent study, we confirmed that mTORC1 promoted mineralized matrix secretion by odontoblasts via the p53 signaling pathway [14]. Additionally, we found that the expression levels of cluster of differentiation 63 (CD63) and apoptosis-linked gene 2-interacting protein (Alix) changed with changes in mTORC1 activity and that predentin was thicker in molars in 6-month-old mice with activated mTORC1. Previous studies have reported that CD63 and Alix are biomarkers of exosomes [15–18]. In developed teeth, predentin is part of secondary dentin, and it is well known that secondary dentin partially originates from DPSC odontoblastic differentiation [19]. Therefore, we hypothesized that mTORC1 may regulate the release of exosomes from odontoblasts to control the odontoblastic differentiation of DPSCs.

It is well accepted that exosomes originate from intraluminal vesicles [20]. Exosomes have been reported to mediate intercellular communication via the biological molecules (i.e., proteins, nucleic acids, lipids, and metabolites) that they carry [21]. The particle size of exosomes is smaller than 200 nm [22]. Because of the significant role of exosomes in intercellular communication, the effect of exosomes on tooth development and dental stem cell differentiation has recently attracted increasing attention. In a recent study, Hertwig’s epithelial tooth sheath cells promoted dental papilla cell differentiation through their exosome-like vesicles in a dental pulp microenvironment [23]. Furthermore, exosomes derived from DPSCs can regulate the angiogenesis of endothelial cells [24] and promote the osteoblastic differentiation of homotypic DPSCs [25]. Jiang et al. reported that exosomes mediate epithelial–mesenchymal crosstalk to promote tooth development [26]. Researchers also found that exosomes that are released from severely inflamed odontoblasts attenuate apoptosis in mildly inflamed neighboring odontoblasts [27]. In conclusion, exosomes are crucial for dental stem cell differentiation and tooth development. However, whether exosomes that are derived from odontoblasts play a critical role in the odontoblastic differentiation of DPSCs is still unknown.

In this study, the objectives were to investigate the roles of mTORC1 in the release of exosomes by odontoblast in order to regulate the odontoblastic differentiation of DPSCs; to this end, mTORC1 was activated and inhibited in vitro and in vivo, and the underlying mechanism was elucidated via microRNA (miRNA) sequencing of exosomes that were derived from odontoblasts. Furthermore, we investigated the possible regulatory mechanism by which DPSCs remain in a quiescent state, and our findings may provide a theoretical basis for the development of strategies to promote dentin-pulp complex regeneration.

## Methods

### Animals

All animal experiments were performed with the approval of the Ethical Committee for Animal Research of Southern Medical University and were conducted according to the state guidelines of the Ministry of Science and Technology of China. A study has shown that DMP1-Cre is expressed in odontoblasts and osteocytes [28]. Dmp1-Cre^+^ (JAX stock 023047) mice and TSC1^f/f^ (129S6/SyEyTac) mice were purchased from Jackson Laboratory (Bar Harbor, USA). The breeding strategies were performed as previously described [14]. To generate TSC1-deficient in odontoblast, we mated TSC1^f/f^ mice with DMP1-Cre^+^ mice to generate DMP1-Cre^+^TSC1^f/+^mice. Then, DMP1-Cre^+^TSC1^f/+^mice were mated with TSC1^f/f^ to obtain male conditional knockout mice (Dmp1-Cre^+^ TSC1^f/f^; CKO) and littermates of the same sex served as controls (Dmp1-Cre^−^TSC1^f/f^; Ctrl). The other offspring genetypes, including DMP1-Cre^+^TSC1^f/+^ and DMP1-Cre^−^TSC1^f/+^, were excluded from analyses, and the number is 36. The random allocation of mice to experimental group (control versus knockout) was driven by Mendelian Inheritance. For each mouse, two different investigators were involved as follows: First investigator (SH) was responsible for the mice generating mice genetype identification; second investigator (HL) was responsible for micro-CT scanning, HE staining and immunofluorescence analyses. For the euthanasia, the mice were firstly anesthetized by 1.5% isoflurane and then were performed dislocation of cervical vertebra. Four 6-month-old CKO mice samples and four 6-month-old Ctrl mice samples were used to analyses in this study. Every mouse is regarded as the single experiment unit, and all the mice were kept in SPF laboratories.

### Micro-CT analysis

Mandibular molars were harvested from 6-month-old mice for micro-CT scanning (Scanco Medical, AG, Switzerland) at 12-µm accuracy. All the specimens were incubated in 4% paraformaldehyde at 4 °C for 24 h. Then, micro-CT scanning was performed. The dentin thickness was measured in sections of mesiobuccal cusps of mandibular first molars. The dentin percentage of mandibular first molars was also analyzed by Scanco software.

### Immunofluorescence and hematoxylin and eosin staining

Mouse mandibular molars were fixed in 4% paraformaldehyde for 48 h and then decalcified in 10% ethylenediaminetetraacetic acid at 4 °C for 1 month. A series of gradient ethanol solutions was used for tissue dehydration, and then, the tissues were embedded in paraffin. Four-micron-thick specimens from first molars were obtained for hematoxylin and eosin (H&E) staining and immunofluorescence, which were performed as previously described [14]. For immunofluorescence, the sections were stained with antibodies against the following proteins: DMP1 (Abcam, UK), COL1 (Proteintech, China), CD63 (Abcam, UK) and Alix (Abcam, UK). The immunofluorescence images were taken by Leica STELLARIS 5 (72 dpi × 72 dpi), LAS X software.

### Cell culture

DPSCs and periodontal ligament stem cells (PDLSCs) were collected from noncarious and periodontally healthy mandibular molars of human patients at the Department of Stomatology, Nanfang Hospital, Southern Medical University. This study was approved by the Ethics Committee of Nanfang Hospital, Southern Medical University (Approval number NFEC-2021–339), and the donors provided informed consent. Briefly, dental pulp and periodontal ligaments were carefully removed from the pulp cavity and root face, respectively. After the dental pulp was dissected into approximately 1-mm^2^ pieces with ophthalmic scissors, the fragments were digested with 3 mg/mL type 1 collagenase (Sigma, USA) at 37 °C for 30 min. Then, the cells were cultured in alpha-MEM supplemented with 10% BSA and 100 U/mL penicillin/streptomycin at 37 °C in 5% CO_2_. When the cells reached 80–90% confluence, the DPSCs and PDLSCs that had clone formation capacity were obtained via a limited dilution method. DPSCs and PDLSCs at passages 3–5 were used for the experiments in this study. The odontoblastic differentiation of DPSCs and PDLSCs was induced with differentiated medium supplemented with 50 μg/mL ascorbic acid and 10 mM β-glycerol phosphate. MDPC23 cell culture was performed as previously described [14]. The MDPC23 cell line, which is an immortalized cell line derived from fetal mouse molar papillae [29, 30], was purchased from Cellcook Biology Company. The MDPC23 cells were cultured in alpha-MEM (Gibco) supplemented with 10% fetal bovine serum (FBS, Gibco) at 37 °C in 5% CO_2_.

### Flow cytometry and oil red O staining

Third-passage DPSCs and PDLSCs were incubated with antibodies against surface markers, including CD29, CD90, CD34 and CD45, at 37 °C for 15 min and then analyzed by flow cytometry.

Oil Red O staining was performed to determine lipid formation during the adipogenic differentiation of DPSCs and PDLSCs. The cells were fixed with 4% paraformaldehyde for 15 min and then washed with PBS 3 times. The cells were stained with Oil Red O staining solution (Cyagen, America) for 10 min, and then, 60% isopropanol was used to differentiate the cells. Lipid droplets were observed under a microscope and photographed.

### DPSC coculture with MDPC23 cells

For the DPSC and MDPC23 coculture experiments, DPSCs were seeded in the bottom chambers of transwell plates with a pore size of 0.4 µm (Corning, USA). Then, MDPC23 cells were labeled with DiO dye according to the manufacturer’s instructions (Beyotime, China). Then, the labeled MDPC23 cells were placed into the upper chambers of the same wells and incubated for 24 h or 7 days. The 0.4-µm transwell allowed the free exchange of molecules, including exosomes, between the DPSCs and MDPC23 cells but prevented cell migration. Notably, MDPC23 cells were transfected with lentivirus to achieve the shRNA-mediated silencing of TSC1 (shTSC1, 5′-GACACACAGAATAGCTATG-3′) in order to activate mTORC1 before they were cocultured with DPSCs. After 24 h, DPSCs were harvested and stained with DAPI (Abcam, UK). The fluorescence images were taken by Olympus BX63 (72 dpi × 72 dpi), Cellsens software.

### MPDC23 cell-derived exosome uptake by DPSCs in vitro

The green fluorescent dye PKH67 was used to label exosomes according to the manufacturer’s instructions (Sigma-Aldrich, USA). Briefly, 0.4 µL in 200 µL Diluent C was added to 10 µg exosomes in 200 *µ*L PBS, and then, the mixture was incubated for 2 min at 24 °C. The excess dye in the mixture was removed by ultracentrifugation at 100,000 × *g* at 4 °C for 70 min to collect the exosomes, which were resuspended in PBS. Then, 10^7^ particles/mL PKH67-labeled exosomes were cocultured with DPSCs at 37 °C for 12 h. Then, the cells were fixed in 4% paraformaldehyde. After the cell nuclei were stained with DAPI (Abcam, UK), the uptake of exosomes was observed by Olympus BX63 (72 dpi × 72 dpi), Cellsens software.

### Immunocytochemistry

Cells were fixed with 4% paraformaldehyde for 30 min at room temperature, and then, they were incubated with 0.5% (v/v) Triton X-100 in PBS for 30 min at room temperature. The cells were blocked with 5% bovine serum albumin (BSA; Biotopped, China) for 30 min and then incubated with primary antibodies against pS6 (Cell Signaling Technology, USA), CD63 (Abcam, UK) and Alix (Abcam, UK) overnight at 4 °C. After extensive washing in PBS, the cells were incubated with a Cy3-labeled goat anti-rabbit IgG antibody. The immunofluorescence images were taken by Olympus BX63 (72 dpi × 72 dpi), Cellsens software.

### Isolation and characterization of exosomes from MDPC23 cells with inhibited or activated mTORC1

shTSC1 and rapamycin were used to activate and inhibit mTORC1 activity, respectively. The methods were performed as previously described [14]. When mTORC1 inactivation or activation, MDPC23 cells were cultured in alpha-MEM supplemented with 10% exosome-free FBS for 24 h or 48 h to obtain conditioned medium. The conditioned medium was differentially centrifuged at 500 × *g* for 10 min, 2000 × *g* for 10 min and 100,00 × g for 30 min at 4 °C, and then, the medium was filtered through a 0.22-µm filter. Next, the medium was ultracentrifuged at 100,000 × *g* at 4 °C for 70 min to collect the exosomes. After being washed and resuspended in PBS, the exosomes were collected again by ultracentrifugation at 100,000 × *g* at 4 °C for 70 min.

Transmission electron microscopy was used to observe the morphology of exosomes, and ZETAVEW (Particle, Germany) was used to analyze the particle size distribution of the exosomes. Western blotting was performed to measure the expression of the protein markers of exosomes CD63 (Abcam, UK) and Alix (Abcam, UK).

### Alizarin red S staining and alkaline phosphatase staining

Alkaline phosphatase (ALP) staining and Alizarin Red S staining, as well as the quantification of Alizarin Red S staining, were performed as previously described [14].

### Western blotting analysis

Lysates of cells were prepared, and western blotting analysis was performed as previously described [14]. Primary antibodies against the following proteins were used: pS6 (Cell Signaling Technology, USA), S6 (Cell Signaling Technology, USA), ALP (Abcam, UK), COL1 (Abcam, UK), GAPDH (Bioworld, China), DMP1 (Bioss, China), ALIX (Bimake, China) and CD63 (Bimake, China).

### qRT‒PCR

Total RNA was extracted from cells, and qRT‒PCR analysis was performed as described previously [31]. Briefly, TRIzol (Takara, Shiga, Japan) was used to extract the total RNA from the cells according to the manufacturer’s instructions. An aliquot of 1000 ng of RNA was reverse transcribed into cDNA using TaKaRa PrimeScript Reverse Transcriptase (TaKaRa, Shiga, Japan). qRT‒PCR was performed with SYBR Premix DimerEraserTM (Takara) using a QuantStudio 5 Real-time PCR system (Applied Biosystems, USA). The sequences of the primers used in the experiments are listed in Table 1. The relative expression of the target genes was calculated using the 2^−∆∆CT^ method.

**Table 1:** Primers used for qRT-PCR

Target gene	Primer sequence
GAPDH	Forward: CAACGTGTCAGTGGTGGACCTG
	Reverse: GTGTCGCTGTTGAAGTCAGAGGAG
ALP	Forward: ACTGGTACTCAGACAACGAGAT
	Reverse: ACGTCAATGTCCCTGATGTTATG
DMP1	Forward: AGCAGTGAGTCCAGCCAAGAGG
	Reverse: AGTTGTGGGGTCGGGGTTATCTC
COL1	Forward: AGTGGTTTGGATGGTGCCAA
	Reverse: GCACCATCATTTCCACGAGC

### miRNA sequencing

miRNA sequencing was performed by Novogene Bioinformatics Technology (Beijing, China). Briefly, total RNA was isolated from the samples by using the exoRNeasy Midi/Max Kit (Qiagen, USA) according to the manufacturer’s protocol. The extracted total RNA was analyzed by an Agilent 2100 Bioanalyzer (Agilent Technologies, USA). After the total RNA was quantified, the sequencing RNA library was constructed with the Small RNA Sample Pre Kit, which included the following steps: addition of a 3’-end linker, addition of a 5’-end linker, reverse transcription, amplification, cDNA library size selection and purification. Qubit 2.0 was used for preliminary quantification, and then, an Agilent 2100 was used to measure the insert size of the library. After qualified library inspection, the library was pooled for Illumina SE50 sequencing according to the requirements of effective concentration and target offline data amount. The sequencing process was controlled by the data collection software provided by Illumina that performed real‐time data analysis.

### Statistical analysis

Statistical analysis of the results was conducted with GraphPad Prism software (GraphPad, San Diego, CA, USA). Comparisons between two groups were made with unpaired *t* tests. All the data are expressed as the mean ± SEM, and the significance level was set to *p* < 0.05 for data from at least three independent samples.

## Results

### mTORC1 activation promoted dentin formation but inhibited exosome release in vivo

**Fig. 1:**
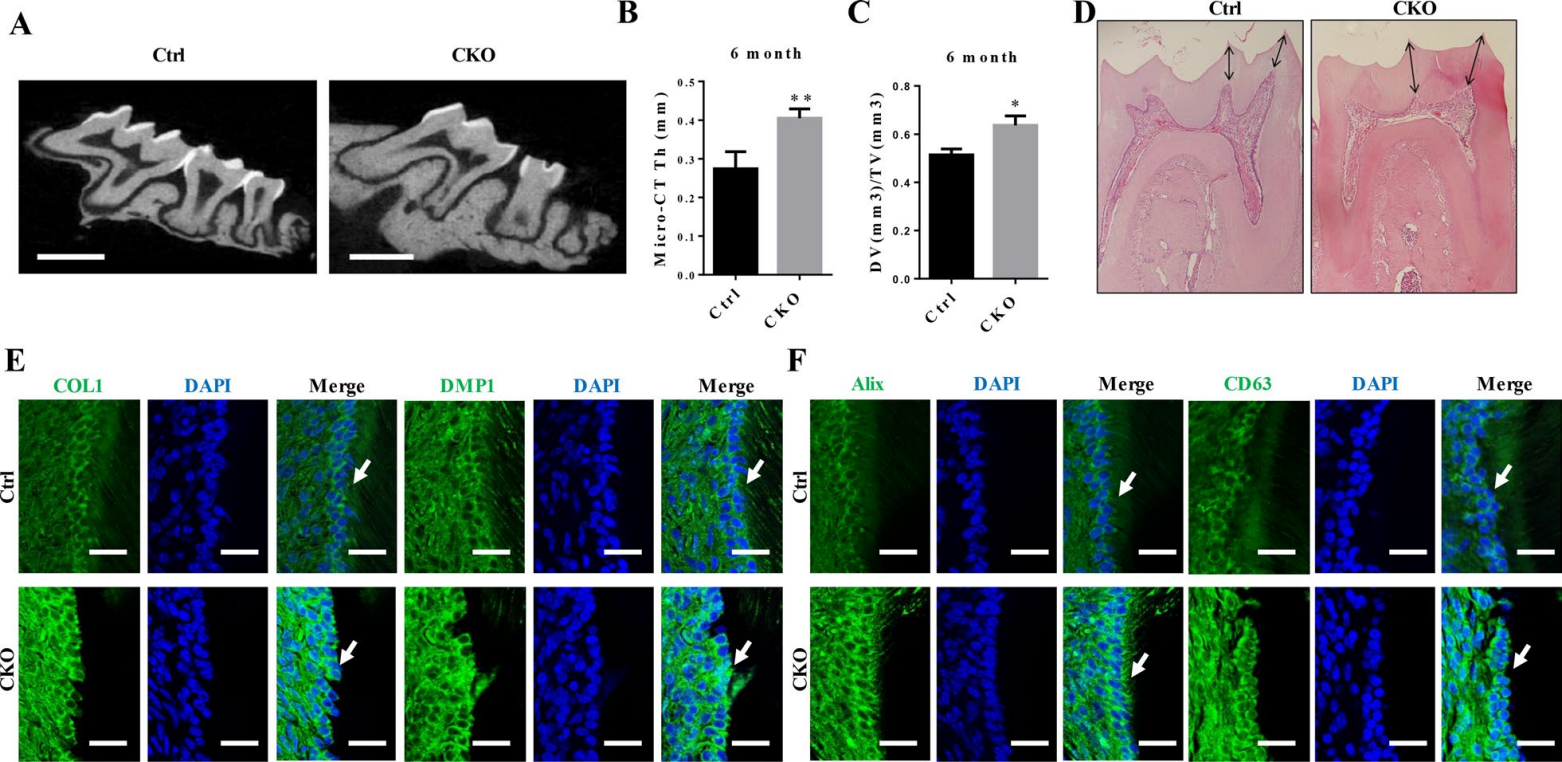
mTORC1 activation promoted dentin formation but inhibited exosome release in vivo. **A** Representative images indicated that CKO mice had significantly thicker dentin in the first and second molars than Ctrl mice (scale bar = 1.0 mm). **B** Dentin thickness in Ctrl (0.2740 ± 0.02209, *N* = 4) mice and CKO mice (0.4048 ± 0.01217, *N* = 4), as determined by micro-CT analysis. **C** Micro-CT analysis of the dentin region in the first molars indicated that the dentin volume/tooth volume (DV/TV) percentages were higher in CKO (0.6362 ± 0.02300, *N* = 4) mice than in Ctrl (0.5138 ± 0.01440, *N* = 4) mice. **D** H&E staining of mandibular first molars from Ctrl mice and CKO mice (scale bar = 200 μm). **E** Immunofluorescence results of the protein expression levels of COL1 and DMP1 in odontoblasts from 1- to 3-month-old CKO mice and Ctrl mice (scale bar = 50 μm). **F** The odontoblasts from 6-month-old CKO mice exhibited stronger staining for CD63 and Alix, which are exosome markers, than those from control mice (scale bar = 50 μm). The values are presented as the means ± SDs. *p < 0.05, **p < 0.01, ***p < 0.001

While studying the role of mTORC1 in dentin formation, we discovered that dentin formation was increased in odontoblasts with mTORC1 overactivation. Micro-CT analysis indicated that the dentin thickness in 6-month-old CKO mice was significantly higher than that in 6-month-old control mice (Fig. 1A, B). Analysis of the dentin volume/tooth volume (DV/TV) in the dentin region showed that the DV/TV of 6-month-old CKO mouse molars was approximately 12% greater than that of control mice (Fig. 1C). In addition, H&E staining showed the same results as micro-CT analysis, and compared to the control mice, the formation of predentin in the CKO mice was increased (Fig. 1D). As shown in Fig. 1E, the levels of mineralization-related proteins (DMP1 and COL1) were dramatically increased in the 6-month-old CKO mice compared with the 6-month-old control mice. Additionally, we serendipitously discovered that the odontoblasts of the 6-month-old CKO mice expressed higher levels of CD63 and Alix, which are markers of exosomes, that those of the control mice (Fig. 1F).

Because the odontoblastic differentiation of DPSCs participates in synthesizing reactionary dentin and the concentrations of exosomes are associated with many physiological and pathological activities, we investigated whether there was a relationship between the abnormal accumulation of exosomes in TSC1-deficient odontoblasts and the odontoblastic differentiation of DPSCs.

### Odontoblastic differentiation of DPSCs was inhibited by coculture with odontoblasts, but this inhibition was reversed by coculture with odontoblasts with activated mTORC1

**Fig. 2:**
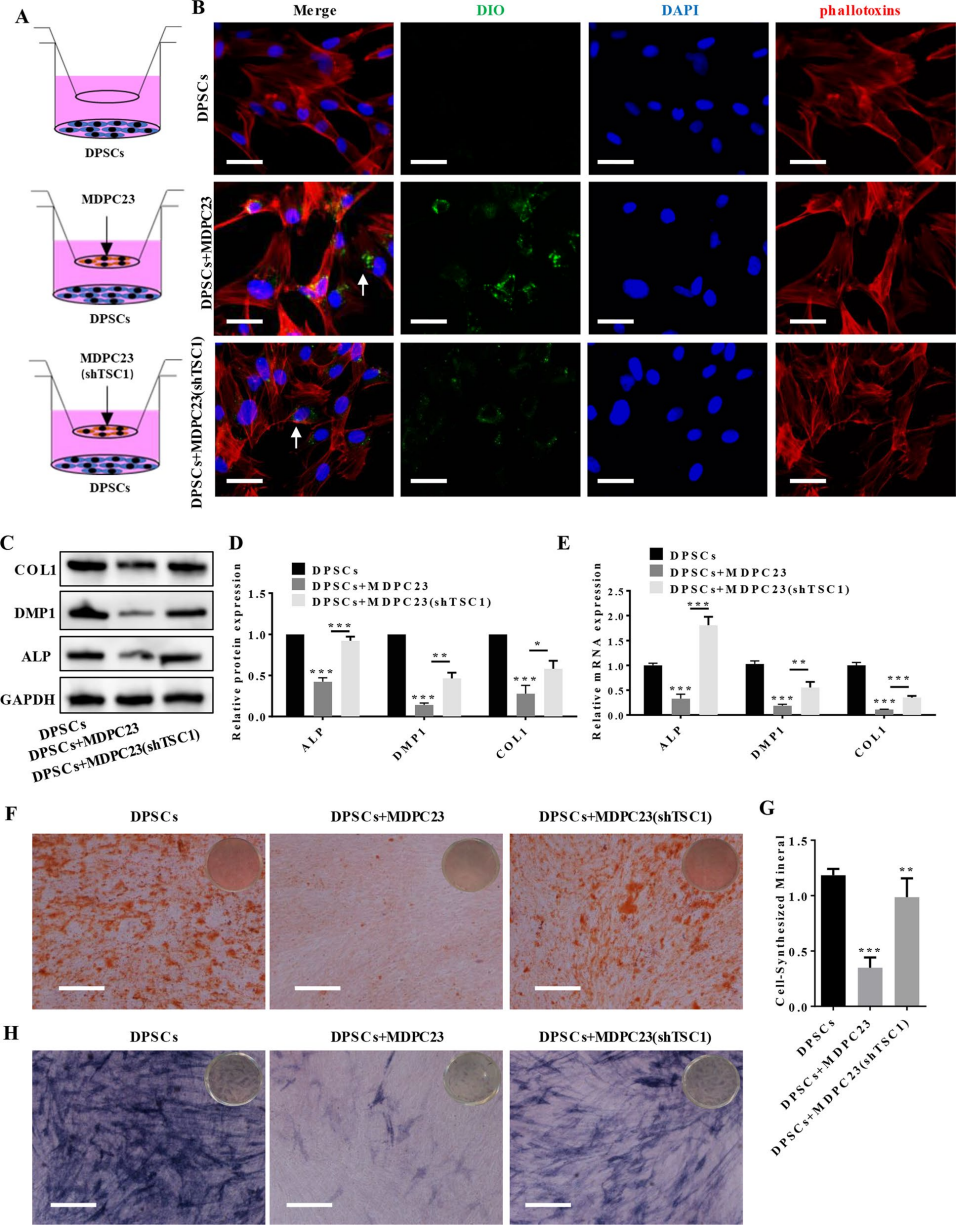
Odontoblastic differentiation of DPSCs was inhibited by coculture with odontoblasts, but this inhibition was reversed by coculture with odontoblasts with activated mTORC1. **A** Schematic diagram showing the method for transwell coculture of DPSCs and MDPC23 cells. **B** MDPC23 cells were labeled with the DiO green fluorescent dye. After being cocultured for 24 h, DPSCs were stained with phallotoxins and DAPI. Exosomes that were derived from MDPC23 cells were successfully taken up by DPSCs, but when mTORC1 was overactivated in the MDPC23 cells, the uptake of exosomes by DPSCs was decreased (scale bar = 50 μm). **C** Western blotting results revealed that MDPC23 cells attenuated the protein expression of DMP1, COL1 and ALP in DPSCs that had been upregulated by mTORC1 activation in MDPC23 cells. Full-length blots are presented in Additional file 1: Fig. S2. **D** Quantitative and statistical analysis of the western blotting results. **E** qRT‒PCR analysis indicated that MDPC23 cells attenuated the mRNA expression of DMP1, COL1 and ALP in DPSCs that had been upregulated by mTORC1 activation in MDPC23 cells (*n* = 3). **F** Alizarin Red S staining showed that the mineralized nodule formation of DPSCs was markedly decreased by coculture with MDPC23 cells, but the inhibition of mineralized nodule formation ability was reversed when mTORC1 was activated in the MDPC23 cells (scale bar = 400 μm). **G** Quantitative and statistical analysis of Alizarin Red S staining by 10% cetylpyridinium chloride. **H** The results of ALP staining (scale bar = 400 μm). The values are presented as the means ± SDs. *p < 0.05, **p < 0.01, ***p < 0.001. *DPSC* Dental pulp stem cells, *ALP* alkaline phosphatase

Primary DPSCs were successfully harvested from dental pulp tissue (Additional file 1: Fig. S1A) and passaged (Additional file 1: Fig. S1B). Alizarin Red S staining and Oil Red O staining showed that induced DPSCs, but not noninduced DPSCs, exhibited mineralized nodules and lipid droplets; these findings indicated the multidirectional differentiation potential of DPSCs (Additional file 1: Fig. S1C, D). The flow cytometry results showed that DPSCs were positive for CD29 and CD90 expression and negative for CD45 and CD34 expression (Additional file 1: Fig. S1E). To simulate the microenvironment of DPSCs and MDPC23 cells in vivo, we utilized a transwell coculture system and activated mTORC1 in vitro to investigate the effect on DPSCs. We cocultured DPSCs with MDPC23 cells using 0.40-*μ*M transwell plates in vitro, as shown in Fig. 2A, in order to simulate the positional relationships between odontoblasts and DPSCs in the microenvironment. MDPC23 cells were labeled with DiO green, which is a fluorescent dye. After being cocultured for 24 h, the MDPC23 cell-derived exosomes were successfully taken up by the DPSCs (Fig. 2B). As shown in Fig. 2B, when mTORC1 was overactivated in MDPC23 cells, the uptake of exosomes by DPSCs decreased. The western blotting and qRT‒PCR results demonstrated that when DPSCs were cocultured with MDPC23 cells in odonto-differentiated medium, the MDPC23 cells reversed the increase in the expression levels of DMP1, COL1 and ALP in the DPSCs that had been upregulated by mTORC1 activation in MDPC23 cells (Fig. 2C–E, Additional file 1: Fig. S2). As shown in Fig. 2F and G, Alizarin Red S staining showed that mineralized nodule formation in the DPSCs was markedly decreased by coculture with MDPC23 cells, but the inhibition of mineralized nodule formation was reversed when mTORC1 was activated in the MDPC23 cells. ALP staining further confirmed these results (Fig. 2H).

### Exosome release by odontoblasts was negatively correlated with mTORC1 activity

**Fig. 3:**
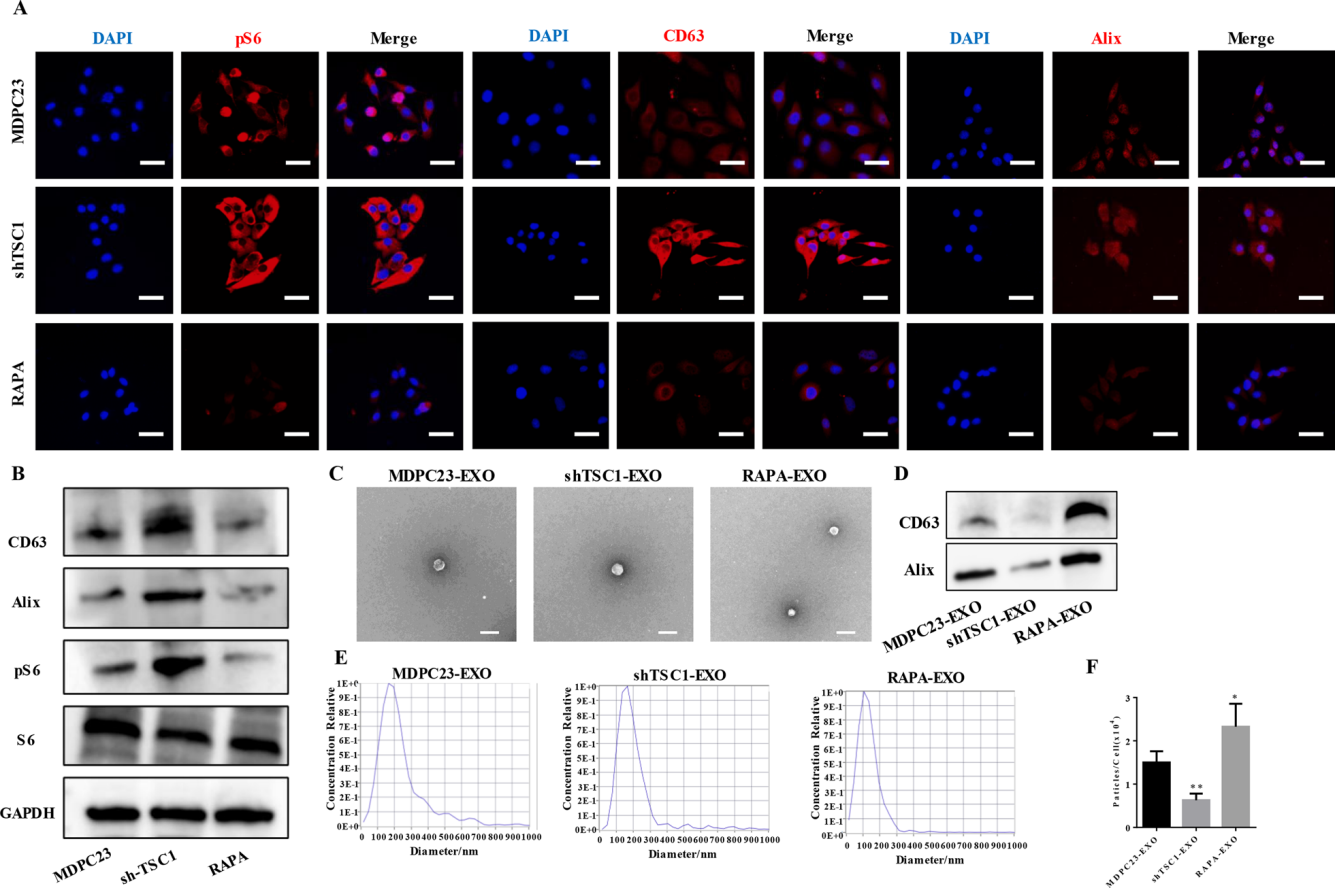
Exosomes release by odontoblasts varied with differences in mTORC1 activity. **A** Immunocytochemistry analysis indicated that the expression levels of pS6 were enhanced in TSC1-deficient MDPC23 cells but markedly decreased in rapamycin-treated cells, and the expression of exosome marker proteins (CD63 and Alix) was positively correlated with mTORC1 activity (scale bar = 50 μm). **B** Western blotting results revealed that the expression of CD63 and Alix was significantly increased in TSC1-deficient MDPC23 cell extracts but was decreased in rapamycin-treated cell extracts. Full-length blots are presented in Additional file 1: Fig. S3. **C** Exosomes that were isolated from MDPC23 cells, TSC1-deficient MDPC23 cells and rapamycin-treated MDPC23 cells were examined by transmission electron microscopy (scale bar = 200 nm). **D** Exosomes that were isolated from cell culture media of MDPC23 cells, TSC1-deficient MDPC23 cells and rapamycin-treated MDPC23 cells were assessed by western blotting. Full-length blots are presented in Additional file 1: Fig. S4. **E** Exosomes that were isolated from MDPC23 cells, TSC1-deficient MDPC23 cells and rapamycin-treated MDPC23 cells were examined by nanoparticle tracking analysis (NTA). **F** The statistical analysis of NTA results. The values are presented as the means ± SDs. *p < 0.05, **p < 0.01, ***p < 0.001

To further confirm the effect of mTORC1 on exosome secretion, we used cell experiments in vitro. Immunocytochemistry analysis indicated that the expression levels of pS6 were enhanced in TSC1-deficient MDPC23 cells but markedly decreased in rapamycin-treated cells, and the expression of exosome marker proteins (CD63 and Alix) was positively correlated with mTORC1 activity (Fig. 3A). Western blotting revealed that the levels of exosome markers, including CD63 and Alix, were greatly increased in TSC1-deficient MDPC23 cell extracts, whereas they were decreased in rapamycin-treated cell extracts (Fig. 3B, Additional file 1: Fig. S3). Next, MDPC23 cells, TSC1-deficient MDPC23 cells and rapamycin-treated MDPC23 cells were cultured in alpha-MEM supplemented with 10% exosome-free FBS for 24 h to obtain conditioned medium, and then, the exosomes were isolated. Examination of the isolated exosomes using transmission electron microscopy showed that the exosomes from control cells, TSC1-deficient cells and rapamycin-treated cells had a similar size and morphology (Fig. 3C). Western blotting results showed that CD63 and Alix could be detected in this group of isolated exosomes (Fig. 3D, Additional file 1: Fig. S4). NTA revealed a sharp decrease in the concentrations of exosomes in the media from TSC1-deficient cells, whereas the concentrations of exosomes in the media from rapamycin-treated cells were significantly increased compared with those of the control group (Fig. 3E, F).

### Exosomes from odontoblasts with mTORC1 activation or mTORC1 inactivation inhibited DPSC differentiation at the same concentration

**Fig. 4:**
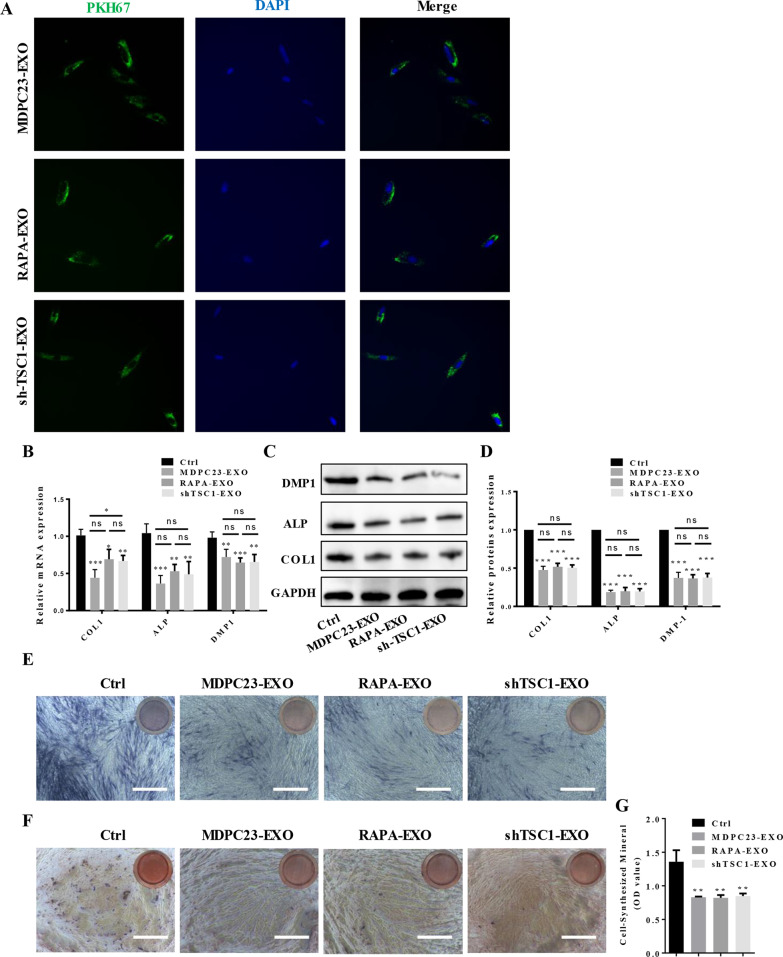
Exosomes from odontoblasts with active mTORC1 and inactive mTORC1 inhibited DPSC odontoblastic differentiation at the same concentration. **A** DPSCs were incubated with PKH67-labeled exosomes from nontreated MDPC23 cells, MDPC23 cells with active mTORC1 and MDPC23 cells with inactive mTORC1 for 24 h. The nuclei of DPSCs were stained with DAPI. The results showed that exosomes that were isolated from these MDPC23 cells could be successfully taken up by DPSCs (scale bar = 100 μm). **B** qRT‒PCR analysis indicated that the expression levels of the odontoblastic differentiation-related genes ALP, COL1 and DMP1 in DPSCs could be significantly inhibited by exosomes derived from MDPC23 cells, regardless of whether mTORC1 was inhibited or activated in the MDPC23 cells (*n* = 3). **C**, **D** DPSCs lysates were analyzed by western blotting. The results indicated that the protein expression levels of ALP, COL1 and DMP1 in DPSCs were dramatically decreased by exosomes that were derived from MDPC23 cells, and there was a nonsignificant difference between exosomes that were isolated from MDPC23 cells with different levels of mTORC1 activity. Full-length blots are presented in Additional file 1: Fig. S5. **E–G** The variation in DPSC mineralization was examined by ALP activity staining and Alizarin Red S staining. The results revealed that compared with differentiated DPSCs with normal mineralization, the activity of ALP **E** and the formation of mineralized nodules **F**, **G** were inhibited by exosomes that were derived from MDPC23 cells, and the effects of exosomes derived from cells with different levels of mTORC1 activity were similar (scale bar = 400 μm). The values are presented as the means ± SDs. *p < 0.05, **p < 0.01, ***p < 0.001. *DPSC* Dental pulp stem cells, *ALP* alkaline phosphatase

To confirm the role of mTORC1 in the release of exosomes by odontoblasts in order to regulate DPSCs odontoblast differentiation, we first collected exosomes from MDPC23 cells, mTORC1-activated MDPC23 cells and mTORC1-inactivated MDPC23 cells and cocultured them with DPSCs. As shown in Fig. 4A, the exosomes were labeled with PKH67, and most DPSCs exhibited strong intracellular green fluorescence. The results showed that the exosomes that were isolated from nontreated, mTORC1-activated and mTORC1-inactivated MDPC23 cells could be successfully taken up by the DPSCs. qRT‒PCR analysis indicated that the expression levels of the odontoblast differentiation-related genes ALP, COL1 and DMP1 in DPSCs could be significantly inhibited by exosomes that were derived from MDPC23 cells, regardless of whether mTORC1 was inhibited or activated in the MDPC23 cells (Fig. 4B). Western blotting results indicated that the protein expression levels of ALP, COL1 and DMP1 in DPSCs were dramatically decreased by exosomes that were derived from MDPC23 cells, and there was a nonsignificant difference between the exosomes collected from MDPC23 cells with different levels of mTORC1 activity (Fig. 4C, D, Additional file 1: Fig. S5). Furthermore, we evaluated the variation in DPSC mineralization by ALP activity staining and Alizarin Red S staining. As expected, the results revealed that compared with differentiated DPSCs with normally mineralization, the activity of ALP (Fig. 4E) and the formation of mineralized nodules (Fig. 4F, G) were inhibited by MDPC23 cell-derived exosomes, and the effects of exosomes derived from cells with different levels of mTORC1 activity were similar.

### mTORC1 could not regulate the contents of exosomes

**Fig. 5:**
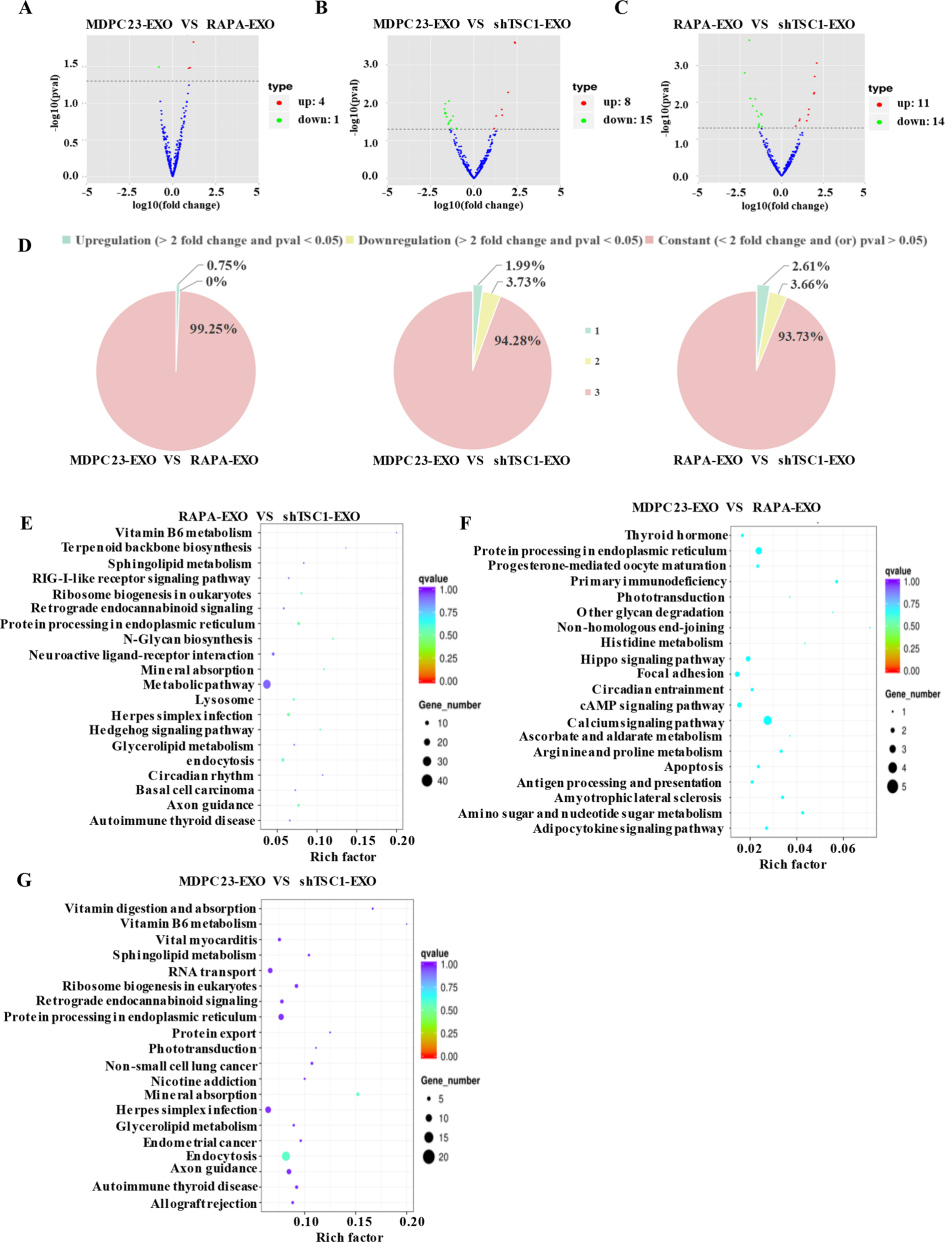
mTORC1 did not regulate the contents of exosomes. **A** Compared to the miRNAs in exosomes from nontreated MDPC23 cells, a total of 402 miRNAs were identified in the exosomes from rapamycin-treated MDPC23 cells; among these miRNAs, 5 were significantly differentially expressed. **B** Compared to the miRNAs in exosomes from nontreated MDPC23 cells, a total of 402 miRNAs were identified in the exosomes from TSC1-deficient MDPC23 cells; among these miRNAs, 23 were significantly differentially expressed. **C** Compared to the miRNAs in exosomes from nontreated MDPC23 cells, a total of 402 miRNAs were identified in the exosomes from TSC1-deficient MDPC23 cells; among these miRNAs, 23 were significantly differentially expressed. **D** The pie chart shows the percentage of unchanged (< twofold change and/or *p* value > 0.05), upregulated (> twofold change and *p* value < 0.05) and downregulated (> twofold change and *p* value < 0.05) miRNAs (MDPC23-EXO vs. RAPA-EXO, MDPC23-EXO vs. shTSC1-EXO and RAPA-EXO vs. shTSC1-EXO). (**E**–**G**) KEGG pathway enrichment analysis showed that the differentially expressed miRNAs mainly participated in the calcium signaling pathway, primary immunodeficiency, protein processing in the endoplasmic reticulum, amino sugar and nucleotide sugar metabolism, and amyotrophic lateral sclerosis, but these differential pathways among the groups were not statistically significant

Exosomes have been reported to mediate intercellular communication via the biological molecules (i.e., proteins, nucleic acids, lipids and metabolites) they carry [21]. Because exosomes that are derived from odontoblasts with different levels of mTORC1 activity have the same effect, we performed miRNA sequencing on exosomes derived from MDPC23 cells that were treated with shTSC1, rapamycin or vehicle to determine whether mTORC1 regulates the content of exosomes. Compared to the miRNAs in exosomes from nontreated MDPC23 cells, a total of 402 miRNAs were identified in the exosomes from rapamycin-treated MDPC23 cells; among these miRNAs, 5 were significantly differentially expressed (Fig. 5A). Compared to the miRNAs in exosomes from nontreated MDPC23 cells, a total of 402 miRNAs were identified in the exosomes from TSC1-deficient MDPC23 cells; among these miRNAs, 23 were significantly differentially expressed (Fig. 5B). Compared to the miRNAs in the exosomes from rapamycin-treated MDPC23 cells, a total of 383 miRNAs were identified in the exosomes from TSC1-deficient MDPC23 cells; among these miRNAs 25 were significantly differentially expressed (Fig. 5C). The constant miRNAs accounted for more than 90% of the total miRNAs, which revealed that although mTORC1 activity varied among these cells, the miRNA contents were mostly indistinguishable (Fig. 5D). Furthermore, Kyoto Encyclopedia of Genes and Genomes (KEGG) pathway enrichment analysis showed that the differentially expressed miRNAs mainly participated in the calcium signaling pathway, primary immunodeficiency, protein processing in the endoplasmic reticulum, amino sugar and nucleotide sugar metabolism, and amyotrophic lateral sclerosis, but these differential pathways among the groups were not statistically significant (Fig. 5E–G). This finding indicates that mTORC1 regulates the release of exosomes by odontoblasts but not the contents of exosomes in order to control the odontoblastic differentiation of DPSCs.

### mTORC1 regulated the release of exosomes by odontoblasts to specifically inhibit DPSC odontoblastic differentiation

**Fig. 6:**
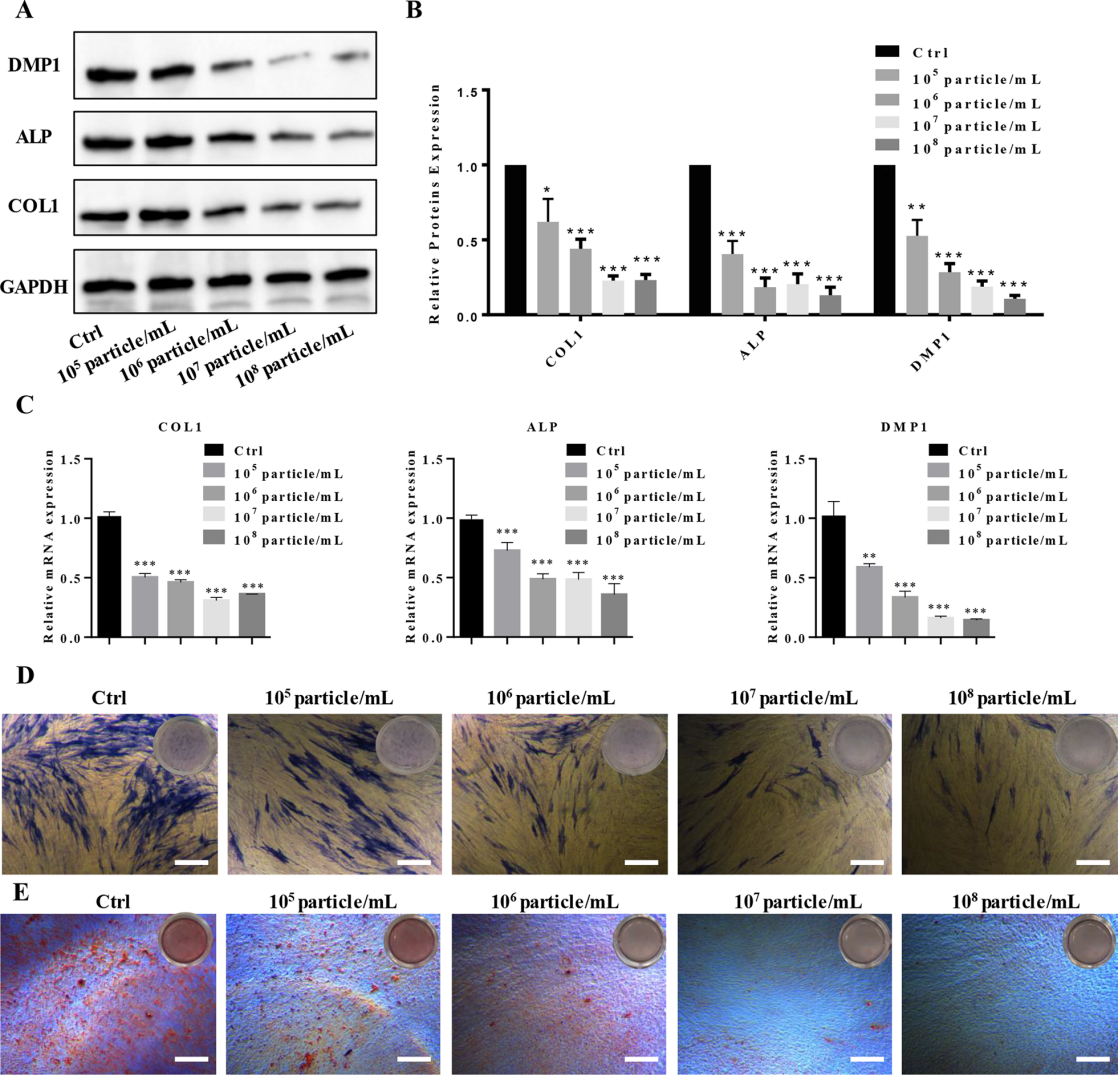
mTORC1 regulated the release of exosomes by odontoblasts to specifically inhibit DPSC odontoblastic differentiation. **A**, **B** DPSCs were incubated with 10^5^ particles/mL, 10^6^ particles/mL, 10^7^ particles/mL and 10^8^ particles/mL exosomes that were isolated from MDPC23 cells, and then, the cell lysates were assessed by western blotting. The results showed that the protein expression levels of the odontoblastic markers ALP, COL and DMP1 gradually decreased as the exosome concentration increased, and 10^8^ particles/mL notably inhibited the expression of ALP, COL and DMP1. Full-length blots are presented in Additional file 1: Figure S6. **C** qRT‒PCR analysis indicated that the miRNA expression levels of ALP, COL and DMP1 were negatively correlated with the concentration of exosomes (*n* = 3). **D** ALP activity staining of DPSCs that were treated with 10^5^ particles/mL, 10^6^ particles/mL, 10^7^ particles/mL and 10^8^ particles/mL exosomes that were isolated from MDPC23 cells (scale bar = 400 μm). **E** The formation of mineralized nodules was examined by Alizarin Red S staining, and the results showed that the formation of mineralized nodules was gradually reduced when the concentration of exosomes was increased, and the formation of mineralized nodules was obviously inhibited at an exosome concentration of 10^8^ particles/mL (scale bar = 400 μm). The values are presented as the means ± SDs. *p < 0.05, **p < 0.01, ***p < 0.001. *DPSCs* Dental pulp stem cells, *ALP* alkaline phosphatase

Next, we investigated the effect of different exosome concentrations released from odontoblasts on the odontoblastic differentiation of DPSCs. DPSCs were incubated with 10^5^ particle/mL, 10^6^ particle/mL, 10^7^ particle/mL and 10^8^ particle/mL exosomes that were isolated from odontoblasts. Western blotting results showed that the protein expression levels of the odontoblastic markers ALP, COL and DMP1 gradually decreased as the exosome concentration increased, and 10^8^ particles/mL inhibited the expression of ALP, COL and DMP1 (Fig. 6A, B, Additional file 1: Fig. S6). qRT‒PCR analysis indicated that the miRNA expression levels of ALP, COL and DMP1 were negatively correlated with the concentration of exosomes (Fig. 6C). These results were further confirmed by ALP activity staining (Fig. 6D). Alizarin Red S staining confirmed that the formation of mineralized nodules was gradually reduced as the concentration of exosomes was increased, and the formation of mineralized nodules was obviously inhibited by a concentration of 10^8^ particles/mL (Fig. 6E).

Furthermore, to confirm the specific negative effect on the odontoblastic differentiation of DPSCs, PDLSCs were stimulated with MDPC23 cell-derived exosomes, and the mineralization ability of the PDLSCs was determined. First, primary PDLSCs were successfully harvested from periodontal ligament tissues (Additional file 1: Fig. S7A). Alizarin Red S staining and Oil Red O staining showed that induced PDLSCs exhibited mineralized nodules and lipid droplets, which indicated the multidirectional differentiation potential of the PDLSCs (Additional file 1: Fig. S7B, C). The flow cytometry results showed that the PDLSCs were positive for CD29 and CD90 expression and negative for CD34 expression (Additional file 1: Fig. S7D). PDLSCs were cocultured with 10^8^ particle/mL exosomes in mineralized-induced medium. qRT‒PCR analysis showed that the mRNA expression level of ALP was not inhibited by the exosomes that were derived from MDPC23 cells and that the mRNA expression levels of COL1 and DMP1 were promoted by the exosomes that were derived from MDPC23 cells (Additional file 1: Fig. S7E). The western blotting results showed a trend similar to that of the qRT‒PCR results (Additional file 1: Fig. S7F). ALP activity staining and Alizarin Red S staining indicated that ALP activity and the formation of mineralized nodules in PDLSCs were not inhibited by exosomes that were derived from MDPC23 cells (Additional file 1: Fig. S7G, H).

## Discussion

It is well known that DPSCs remain quiescent and will not differentiate into odontoblasts under physiological conditions, but the regulatory mechanism remains undefined. In this study, we found that mTORC1 regulated the release of exosomes by odontoblasts to inhibit the odontoblastic differentiation of DPSCs. Furthermore, the results confirmed that mTORC1 regulates only exosome release and not exosomal contents.

In our previous research, it was confirmed that mTORC1 could control odontoblast mineralization and promote dentin formation [14]. However, the increase in dentin thickness and predentin in adult mice depends not only on odontoblasts but also on the odontoblastic differentiation of DPSCs [19]. In this study, we serendipitously found that the expression levels of the exosome markers CD63 and Alix were increased in TSC1-knockdown odontoblasts in vivo. Exosomes play an important role in mediating intercellular communication. Therefore, based on the in vivo results, we tried to determine whether mTORC1 regulates the release of exosomes by odontoblasts in order to control the odontoblastic differentiation of DPSCs. First, we cocultured DPSCs with MDPC23 cells in vitro to mimic their spatial and positional relationships. The results revealed that mTORC1 activation in odontoblasts reversed the inhibition of DPSC odontoblastic differentiation when cocultured with these odontoblasts. Previously, Zhang et al. also used a coculture model to confirm that exosomes from Hertwig’s epithelial root sheath cells were endocytosed by dental papilla cells [23]. In this study, we compared the effects of exosomes that were secreted by MDPC23 cells with different levels of mTORC1 activity on the odontoblastic differentiation of DPSCs and found that exosomes that were secreted by MDPC23 cells with different levels of mTORC1 activity exerted similar effects on the odontoblastic differentiation of DPSCs and limited nodule formation by DPSCs. Additionally, our study confirmed that the release of exosomes by odontoblasts could be promoted by inhibiting mTORC1 activity, and mTORC1 overactivation could decrease the release of exosomes by odontoblasts, showing that the release of exosomes in odontoblasts could be regulated by mTORC1 activity.

In addition, studies have shown that the miRNAs that are carried by exosomes can be used in the diagnosis and treatment of diabetes, breast cancer, myocardial infarction, lung injury and other diseases [32–35]. Exosomes mainly rely on the miRNAs they carry to perform their functions [36]. Thus, in our study, miRNA sequencing analysis was performed on exosomes that were secreted by MDPC23 cells with different levels of mTORC1 activity; the results indicated that the majority of the miRNA content was homogeneous. The differentially expressed miRNAs (miR-880-3p, miR-96-5p, miR-133c, etc.) that were identified bioinformatics analysis mainly function in germ cell development [37], osteoclastic differentiation [38], cancer [39], and proliferation and migration of fibroblasts. However, KEGG enrichment was further performed on the differentially expressed miRNAs, and the results showed that the differential activation of these pathways among these cell types was not statistically significant. Previously, it was reported that mTORC1 could regulate exosome release, but not exosome content, in response to changes in physiological conditions [9], which is consistent with our findings. Although the majority of miRNAs did not exhibit obvious changes in expression, further protein sequencing analysis of exosomes will be necessary to reveal the regulatory mechanism for these processes.

It is well known that stem cell activities are controlled by the external microenvironment [40]. The mechanism by which ASCs, including DPSCs, maintain quiescence remains unknown. Recently, a study reported that Wnt4 produced by muscle fibers maintains muscle stem cell quiescence through RhoA [41]. In this study, we found that exosomes that were derived from odontoblasts inhibited the odontoblastic differentiation of DPSCs, and the inhibitory effect was positively correlated with exosome concentration. When DPSCs were cocultured with 10^7^ particle/mL exosomes, they almost did not differentiate into odontoblasts and remained quiescent. To further confirm that exosomes that are produced by odontoblasts exert a specific effect on DPSCs, we stimulated PDLSCs with odontoblast-derived exosomes under the same conditions. The results revealed that the osteoblastic differentiation of PDLSCs was not inhibited by exosomes derived from odontoblasts. DPSCs have been reported to release exosomes to promote odontoblastic differentiation via the miRNAs that they carry [42]. Our study indicated that mTORC1 regulates the release of exosomes by odontoblasts to inhibit the odontoblastic differentiation of DPSCs and maintain DPSC quiescence.

To date, many studies have elucidated the mechanisms by which exosomes are released. Exosomes are derived from intraluminal vesicles (ILVs) in multivesicular bodies (MVBs) [17]. It has been reported that the fusion of MVBs with the plasma membrane to release exosomes is controlled by two small GTPases of the Rab family, namely, Rab27A and Rab27B [43]. A recent study demonstrated that MBVs can fuse with lysosomes and be protected from degradation, and exosomes are then released by lysosomal exocytosis [44]. In addition, scutellarin activates autophagy by acting on the PI3K/PENT/Akt pathway, which upregulates the expression of Rab8a and promotes the release of exosomes [45]. Regarding the mechanism by which mTORC1 regulates the release of exosomes, mTORC1 regulates the fusion of MVBs with the plasma membrane through Rab27A in HeLa cells [9]. However, how mTORC1 regulates the release of exosomes in odontoblasts is unclear, and whether mTORC1 also regulates exosome release through Rab27A needs to be further explored.

In recent years, exosomes have increasingly attracted attention in the field of targeted drug delivery. One of the key goals of engineering exosomes for targeted drug delivery is increasing exosome production [46]. In this study, we found that rapamycin, which is an mTORC1-specific inhibitor, could promote the release of exosomes by odontoblasts, which may provide a new approach to enhance exosome production. In addition, another major technical limitation for targeted drug delivery is the ability of exosomes to specifically recognize recipient cells. Cells take up exosomes by a variety of endocytic pathways, including caveolin-mediated uptake, micropinocytosis and lipid raft-mediated internalization [47]. Exosomes that are derived from DPSCs can be endocytosed by DPSCs through a caveolar endocytic mechanism. In this study, the mechanism by which DPSCs take up the exosomes derived from odontoblasts remains undefined. It will be important to determine the specific recognition receptor, which could provide a new method for targeted drug delivery to DPSCs.

## Conclusion

In summary, our study confirms that mTORC1 regulates only the number of exosomes released, but not the contents of these exosomes, from odontoblasts to inhibit the odontoblastic differentiation of DPSCs. Additionally, this study reveals that odontoblasts release exosomes to maintain DPSC quiescence. These findings provide new insights that enhance the understanding of the mechanism underlying DPSC odontoblastic differentiation in the microenvironment and will provide a new approach to enhance exosome production.

## Supplementary Information

**Additional file 1.** Supplementary Figures.

## Data Availability

The data have been stored at Gene Expression Omnibus database and can be visited at https://www.ncbi.nlm.nih.gov/geo/query/acc.cgi?&acc=GSE224861.

## Abbreviations

DPSCs: Dental pulp stem cells
CKO: Conditional knockout
ASC: Adult stem cells
TGF-β: Transforming growth factor
TNF-α: Tumor necrosis factor-α
LPS: Lipopolysaccharide
mTOR: Mechanistic target of rapamycin
mTORC1: Mechanistic target of rapamycin complex 1
mTORC2: Mechanistic target of rapamycin complex 2
TSC1: Tuberous sclerosis complex 1
TSC2: Tuberous sclerosis complex 2
RHEB: Ras homolog enriched in brain
S6K1: S6 kinase 1
pS6: Phospho-S6
CD63: Cluster of differentiation 63
Alix: Apoptosis-linked gene 2-interacting protein
miRNA: Micro-RNA
H&E: Hematoxylin and eosin
PDLSCs: Periodontal ligament stem cells
FBS: Fetal bovine serum
BSA: Bovine serum albumin
EDTA: Ethylenediaminetetraacetic acid
shTSC1: Lentivirus for shRNA-mediated silencing of TSC1
DMP1: Dentin matrix protein 1
COL1: Type 1 collagen
ALP: Alkaline phosphatase
DV/TV: Dentin volume/tooth volume
KEGG: Kyoto Encyclopedia of Genes and Genomes
ILVs: Intraluminal vesicle
MVBs: Multivesicular bodies

## References

[1] Rezai RM, Wise GE, Brooks H (2013). Activation of proliferation and differentiation of dental follicle stem cells (DFSCs) by heat stress. Cell Prolif.

[2] Tsutsui TW (2020). dental pulp stem cells: advances to applications. Stem Cells Cloning.

[3] Tao Z, Barker J, Shi SD (2010). Steady-state kinetic and inhibition studies of the mammalian target of rapamycin (mTOR) kinase domain and mTOR complexes. Biochemistry-US.

[4] Inoki K, Zhu T, Guan K (2003). TSC2 mediates cellular energy response to control cell growth and survival. Cell.

[5] Inoki K, Li Y, Zhu T (2002). TSC2 is phosphorylated and inhibited by Akt and suppresses mTOR signalling. Nat Cell Biol.

[6] Saxton RA, Sabatini DM (2017). mTOR signaling in growth, metabolism, and disease. Cell.

[7] Hay N, Sonenberg N (2004). Upstream and downstream of mTOR. Genes Dev.

[8] Laplante M, Sabatini DM (2012). mTOR signaling in growth control and disease. Cell.

[9] Zou W, Lai M, Zhang Y (2019). Exosome release is regulated by mTORC1. Adv Sci (Weinh).

[10] Fan SJ, Kroeger B, Marie PP (2020). Glutamine deprivation alters the origin and function of cancer cell exosomes. EMBO J.

[11] Tanaka Y, Sonoda S, Yamaza H (2018). Suppression of AKT-mTOR signal pathway enhances osteogenic/dentinogenic capacity of stem cells from apical papilla. Stem Cell Res Ther.

[12] Huang B, Wang Y, Wang W (2015). mTORC1 prevents preosteoblast differentiation through the notch signaling pathway. Plos Genet.

[13] Kim JK, Baker J, Nor JE (2011). mTor plays an important role in odontoblast differentiation. J Endod.

[14] Luo X, Yin J, Miao S (2021). mTORC1 promotes mineralization via p53 pathway. FASEB J.

[15] Pols MS, Klumperman J (2009). Trafficking and function of the tetraspanin CD63. Exp Cell Res.

[16] Beinert T, Münzing S, Possinger K (2000). Increased expression of the tetraspanins CD53 and CD63 on apoptotic human neutrophils. J Leukoc Biol.

[17] Hessvik NP, Llorente A (2018). Current knowledge on exosome biogenesis and release. Cell Mol Life Sci.

[18] Hua S, Bartold PM, Gulati K (2021). Periodontal and dental pulp cell-derived small extracellular vesicles: a review of the current status. Nanomaterials (Basel).

[19] Bleicher F (2014). Odontoblast physiology. Exp Cell Res.

[20] Kalluri R (2016). The biology and function of exosomes in cancer. J Clin Invest.

[21] Raposo G, Stahl PD (2019). Extracellular vesicles: a new communication paradigm?. Nat Rev Mol Cell Biol.

[22] Kalluri R, LeBleu VS (2020). The biology, function, and biomedical applications of exosomes. Science.

[23] Zhang S, Yang Y, Jia S (2020). Exosome-like vesicles derived from Hertwig's epithelial root sheath cells promote the regeneration of dentin-pulp tissue. Theranostics.

[24] Zhou H, Li X, Yin Y (2020). The proangiogenic effects of extracellular vesicles secreted by dental pulp stem cells derived from periodontally compromised teeth. Stem Cell Res Ther.

[25] Xie L, Guan Z, Zhang M (2020). Exosomal circLPAR1 promoted osteogenic differentiation of homotypic dental pulp stem cells by competitively binding to hsa-miR-31. Biomed Res Int.

[26] Jiang N, et al. Exosomes mediate epithelium-mesenchyme crosstalk in organ development. ACS Nano. 2017;11(8):7736–46. 10.1021/acsnano.7b01087 PMC5634743 28727410

[27] Wang HS, Yang FH, Wang YJ (2019). Odontoblastic exosomes attenuate apoptosis in neighboring cells. J Dent Res.

[28] Lu Y, Xie Y, Zhang S (2007). DMP1-targeted Cre expression in odontoblasts and osteocytes. J Dent Res.

[29] Hanks CT, Sun ZL, Fang DN (1998). Cloned 3T6 cell line from CD-1 mouse fetal molar dental papillae. Connect Tissue Res.

[30] Hanks CT, Fang D, Sun Z (1998). Dentin-specific proteins in MDPC-23 cell line. Eur J Oral Sci.

[31] Ma D, Yu H, Xu S (2018). Stathmin inhibits proliferation and differentiation of dental pulp stem cells via sonic hedgehog/Gli. J Cell Mol Med.

[32] de Couto G, Gallet R, Cambier L (2017). Exosomal MicroRNA transfer into macrophages mediates cellular postconditioning. Circulation.

[33] Khalaj K, Figueira RL, Antounians L (2020). Systematic review of extracellular vesicle-based treatments for lung injury: are EVs a potential therapy for COVID-19?. J Extracell Vesicles.

[34] Mori MA, Ludwig RG, Garcia-Martin R (2019). Extracellular miRNAs: from biomarkers to mediators of physiology and disease. Cell Metab.

[35] Lakshmi S, Hughes TA, Priya S (2021). Exosomes and exosomal RNAs in breast cancer: a status update. Eur J Cancer.

[36] Sun Z, Yang S, Zhou Q (2018). Emerging role of exosome-derived long non-coding RNAs in tumor microenvironment. Mol Cancer.

[37] Ota H, Ito-Matsuoka Y, Matsui Y (2019). Identification of the X-linked germ cell specific miRNAs (XmiRs) and their functions. PLoS ONE.

[38] Russo R, Zito F, Lampiasi N (2021). MiRNAs expression profiling in Raw26.47 macrophages after Nfatc1-knockdown elucidates potential pathways involved in osteoclasts differentiation. Biology (Basel).

[39] Qin WY, Feng SC, Sun YQ (2020). MiR-96-5p promotes breast cancer migration by activating MEK/ERK signaling. J Gene Med.

[40] Clevers H, Loh KM, Nusse R (2014). Stem cell signalling. An integral program for tissue renewal and regeneration: Wnt signaling and stem cell control. Science.

[41] Eliazer S, Muncie JM, Christensen J (2019). Wnt4 from the niche controls the mechano-properties and quiescent state of muscle stem cells. Cell Stem Cell.

[42] Hu X, Zhong Y, Kong Y (2019). Lineage-specific exosomes promote the odontogenic differentiation of human dental pulp stem cells (DPSCs) through TGFβ1/smads signaling pathway via transfer of microRNAs. Stem Cell Res Ther.

[43] Kowal J, Tkach M, Théry C (2014). Biogenesis and secretion of exosomes. Curr Opin Cell Biol.

[44] Buratta S, Tancini B, Sagini K (2020). Lysosomal exocytosis, exosome release and secretory autophagy: the autophagic- and endo-lysosomal systems go extracellular. Int J Mol Sci.

[45] Hu SQ, Zou YP, Jiang YQ (2022). Scutellarin-mediated autophagy activates exosome release of rat nucleus pulposus cells by positively regulating Rab8a via the PI3K/PTEN/Akt pathway. Cell Biol Int.

[46] Hao Y, Song H, Zhou Z (2021). Promotion or inhibition of extracellular vesicle release: emerging therapeutic opportunities. J Control Release.

[47] Mulcahy LA, Pink RC, Carter DR (2014). Routes and mechanisms of extracellular vesicle uptake. J Extracell Vesicles.

