# Supplementary material for: Odontoblasts release exosomes to regulate the odontoblastic differentiation of dental pulp stem cells
Source: Stem Cell Res Ther. 2023 Jul 8;14:176. doi: 10.1186/s13287-023-03401-9 (PMC10329399; doi:10.1186/s13287-023-03401-9)
Supplement: Supplementary file 1 — Additional file 1. Supplementary Figures. [file 13287_2023_3401_MOESM1_ESM.docx]

# Odontoblasts release exosomes to regulate the odontoblastic differentiation of dental pulp stem cells

Xinghong Luo^1#^, Weiqing Feng^2#^, Shijiang Huang^3#^，Shenghong Miao^2^, Tao Jiang^1^, Qian Lei^1^, Jingyao Yin^2^, Sheng Zhang^3^, Xiaochun Bai^3^, Chunbo Hao^5^, Weizhong Li^4*^, Dandan Ma^1*^

^1^Department of Endodontics, Stomatological Hospital, School of Stomatology, Southern Medical University, Guangzhou, China

^2^College of Stomatology, Southern Medical University, Guangzhou, China

^3^Department of Cell Biology, School of Basic Medical Science, Southern Medical University, Guangzhou, China

^4^Department of Stomatology, Nanfang Hospital, Southern Medical University, Guangzhou, China

^5^Department of Stomatology, Hainan General Hospital, Hainan Affiliated Hospital of Hainan Medical University, Haikou, Hainan, China

***Corresponding author:** Dandan Ma, Department of Endodontics, Stomatological Hospital, Southern Medical University, No 366 Jiangnan Avenue South, Guangzhou, 510280, Guangdong, China. Email: [mdd@smu.edu.cn](mailto:mdd@smu.edu.cn); Weizhong Li, Department of stomatology, Nangfang Hospital, Southern Medical University, No.1838 North Guangzhou Avenue, Guangzhou, People’s Republic of China, CN 510515. Email: [gzliwz@126.com](mailto:gzliwz@126.com).

^#^ Xinghong Luo, Weiqing Feng and Shijiang Huang contributed equally to this work.

**
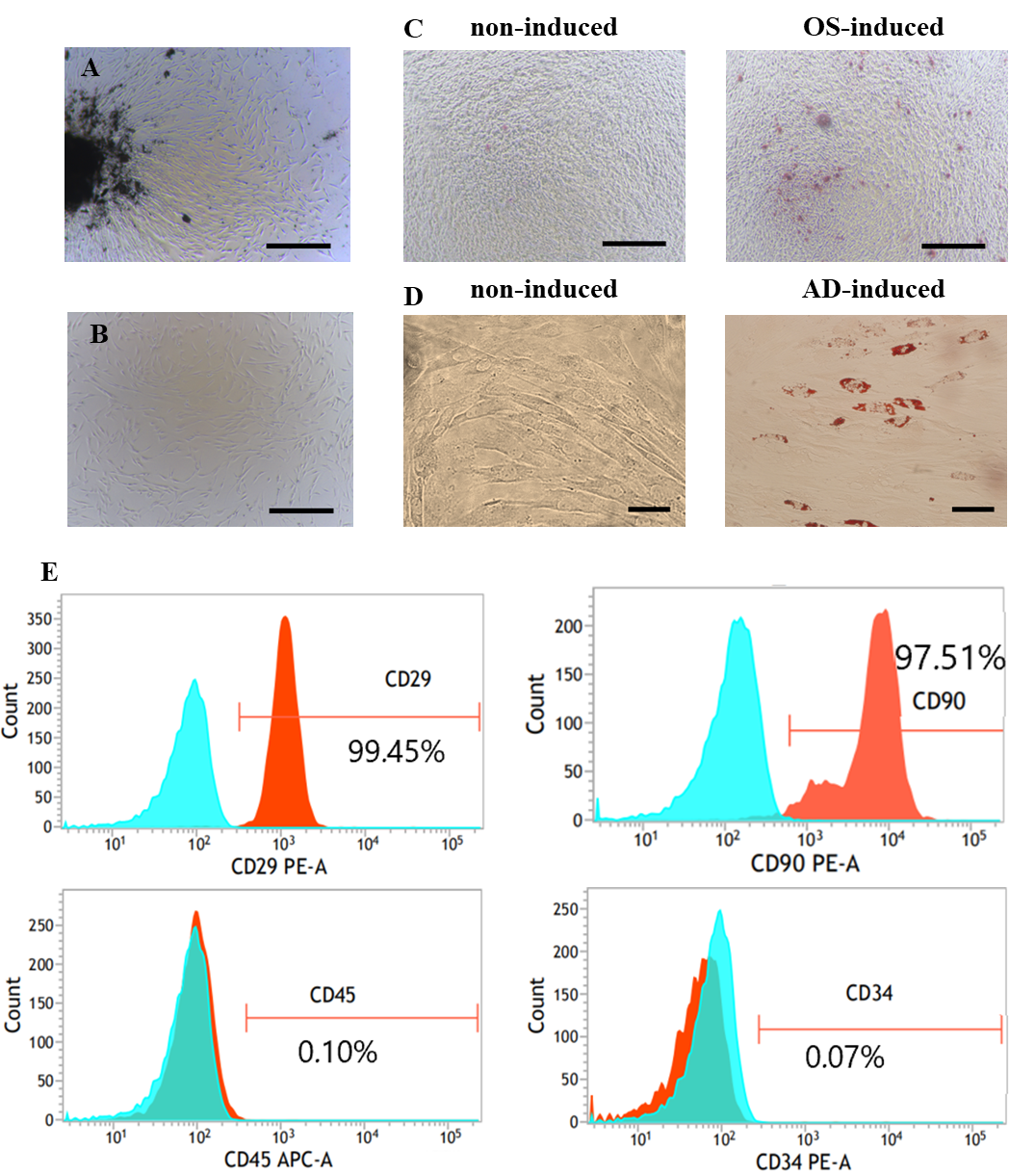
**

**Supplementary Figure 1.** Identification of DPSCs. (A): Primary cultured DPSCs (scale bar = 400 *μ*m). (B): Passaged DPSCs (scale bar = 400 *μ*m). (C): Alizarin Red S showed that the DPSCs that were induced to undergo osteoblastic differentiation exhibited mineralized nodules, but mineralized nodules were not present in the noninduced DPSCs (scale bar = 200 *μ*m). (D) The adipogenic differentiation of cultured DPSCs was assessed by Oil Red O staining, and the results showed the formation of clustered lipid droplets in the induced group but not in the noninduced group (scale bar = 50 *μ*m). (E) Surface marker expression of DPSCs was measured by flow cytometric analysis. DPSCs were positive for CD29 and CD90 expression and negative for CD45 and CD34 expression.

**
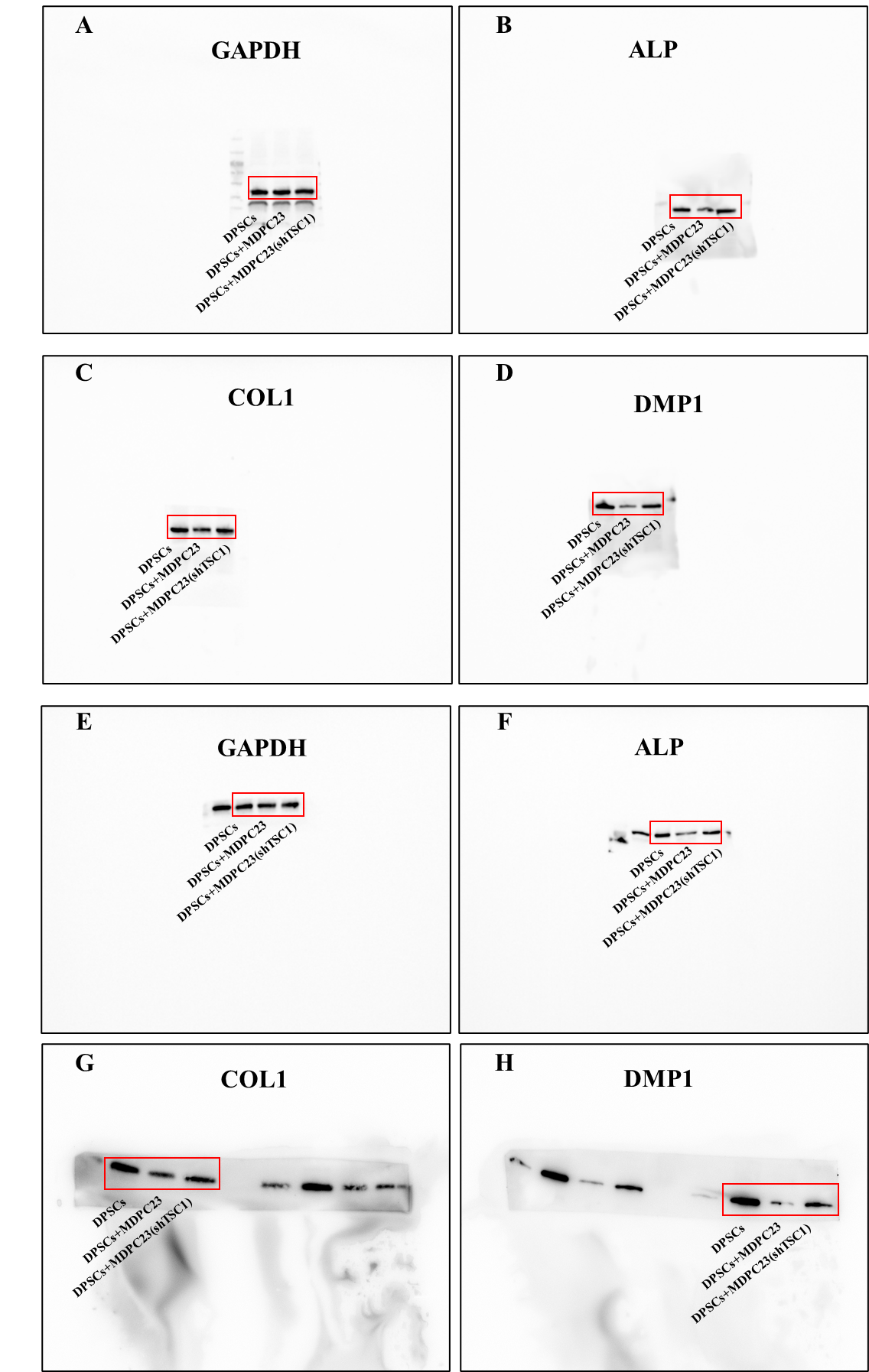
**

**Supplementary Figure 2. A-D：The uncropped western blotting images of Figure 2C and the red lines were used to indicate where they were cropped.** **E-H：The images of Figure 2C repeat experiments，which weren’t presented in manuscript.**

**
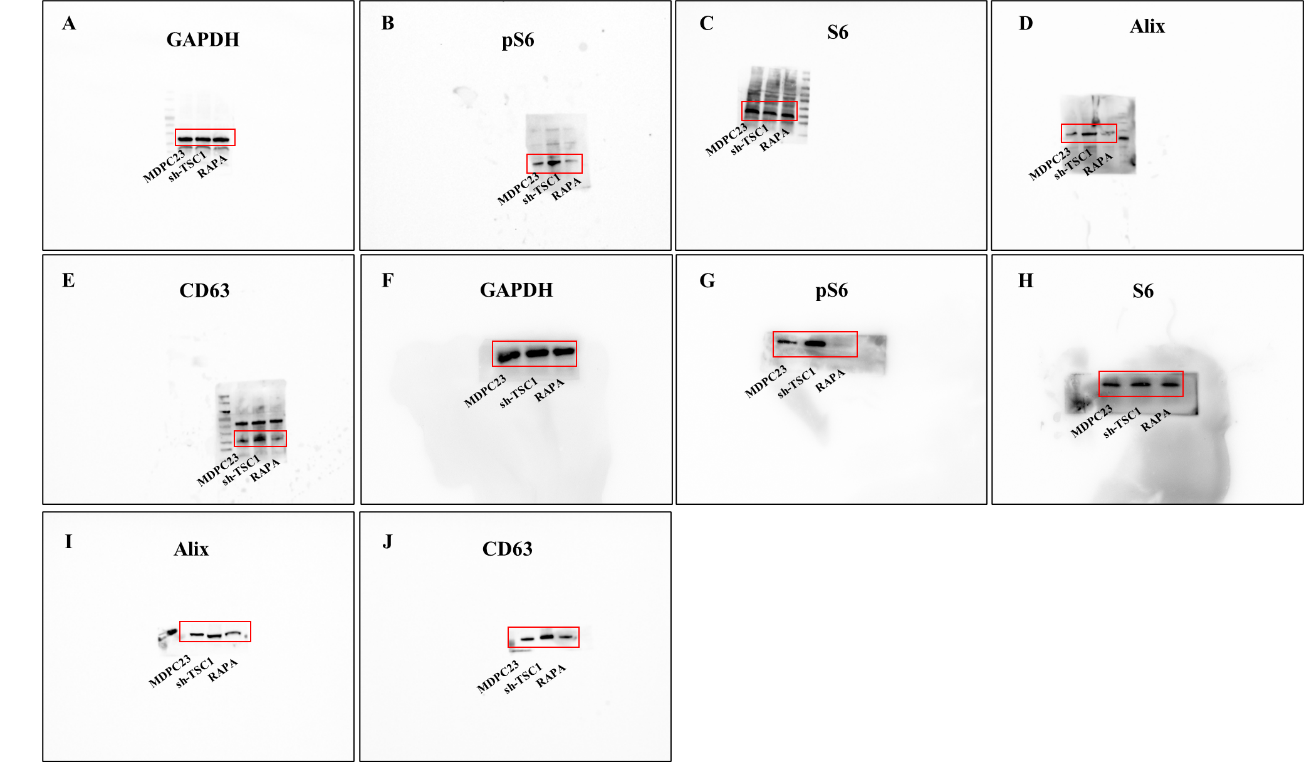
**

**Supplementary Figure 3. A-E: The uncropped western blotting images of Figure 3B and the red lines were used to indicate where they were cropped. F-J: The images of Figure 3B repeat experiments，which weren’t presented in manuscript.**

**
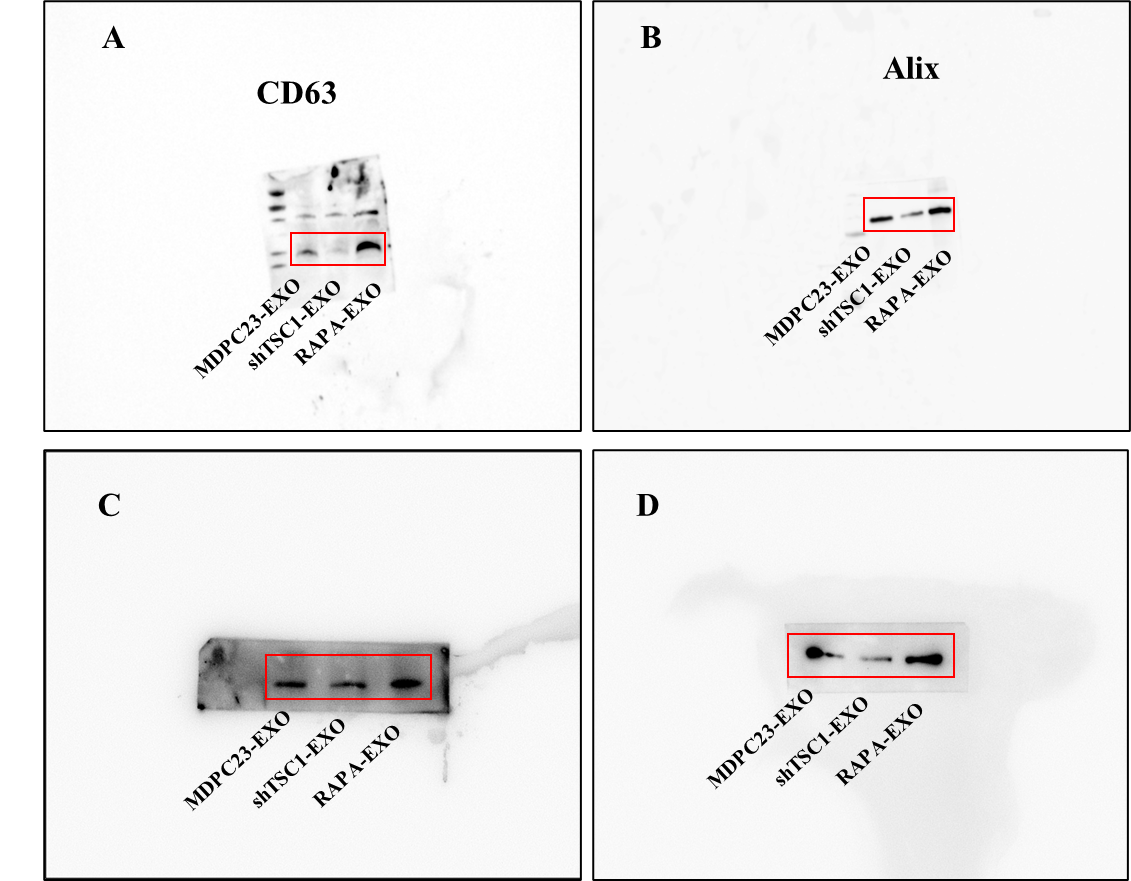
**

**Supplementary Figure 4. A-B: The uncropped western blotting images of Figure 3D and the red lines were used to indicate where they were cropped. F-J: The images of Figure 3D repeat experiments，which weren’t presented in manuscript.**

**
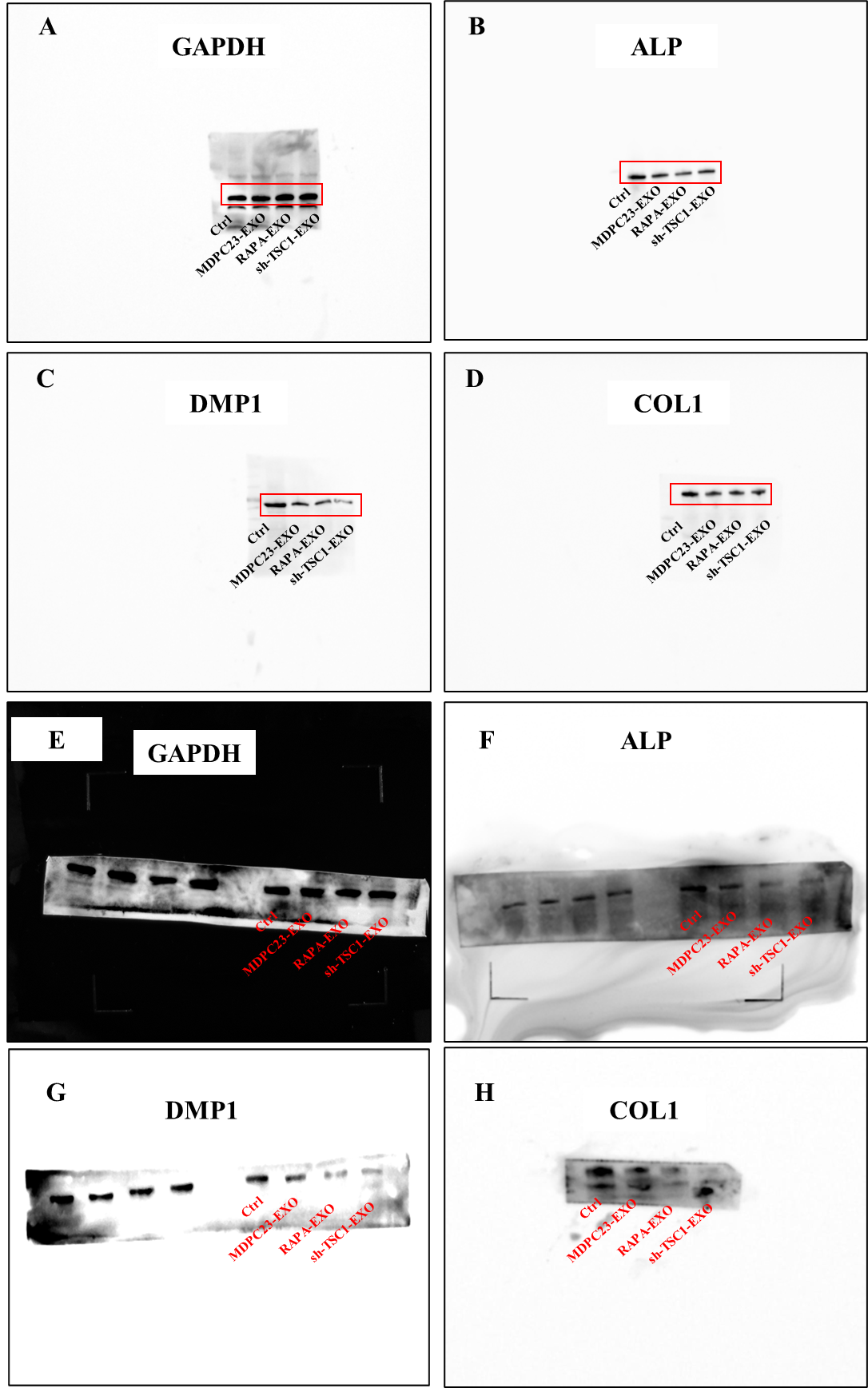
**

**Supplementary Figure 5. A-D: The uncropped western blotting images of Figure 4C and the red lines were used to indicate where they were cropped. E-H：The images of Figure 4C repeat experiments，which weren’t presented in manuscript.**

**
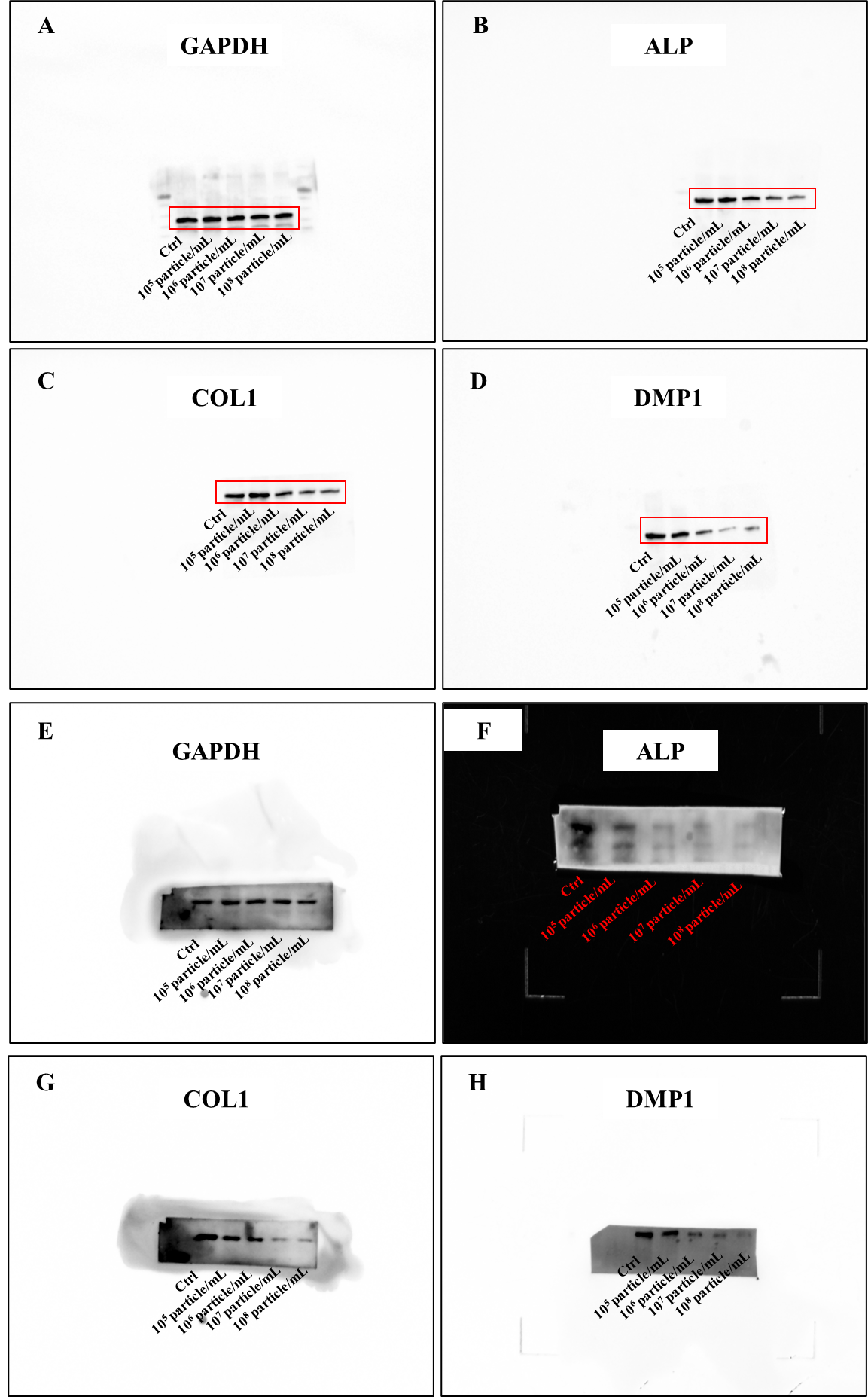
**

**Supplementary Figure 6. A-D: The uncropped western blotting images of Figure 6A and the red lines were used to indicate where they were cropped. E-H：The images of Figure 6A repeat experiments，which weren’t presented in manuscript.**

**
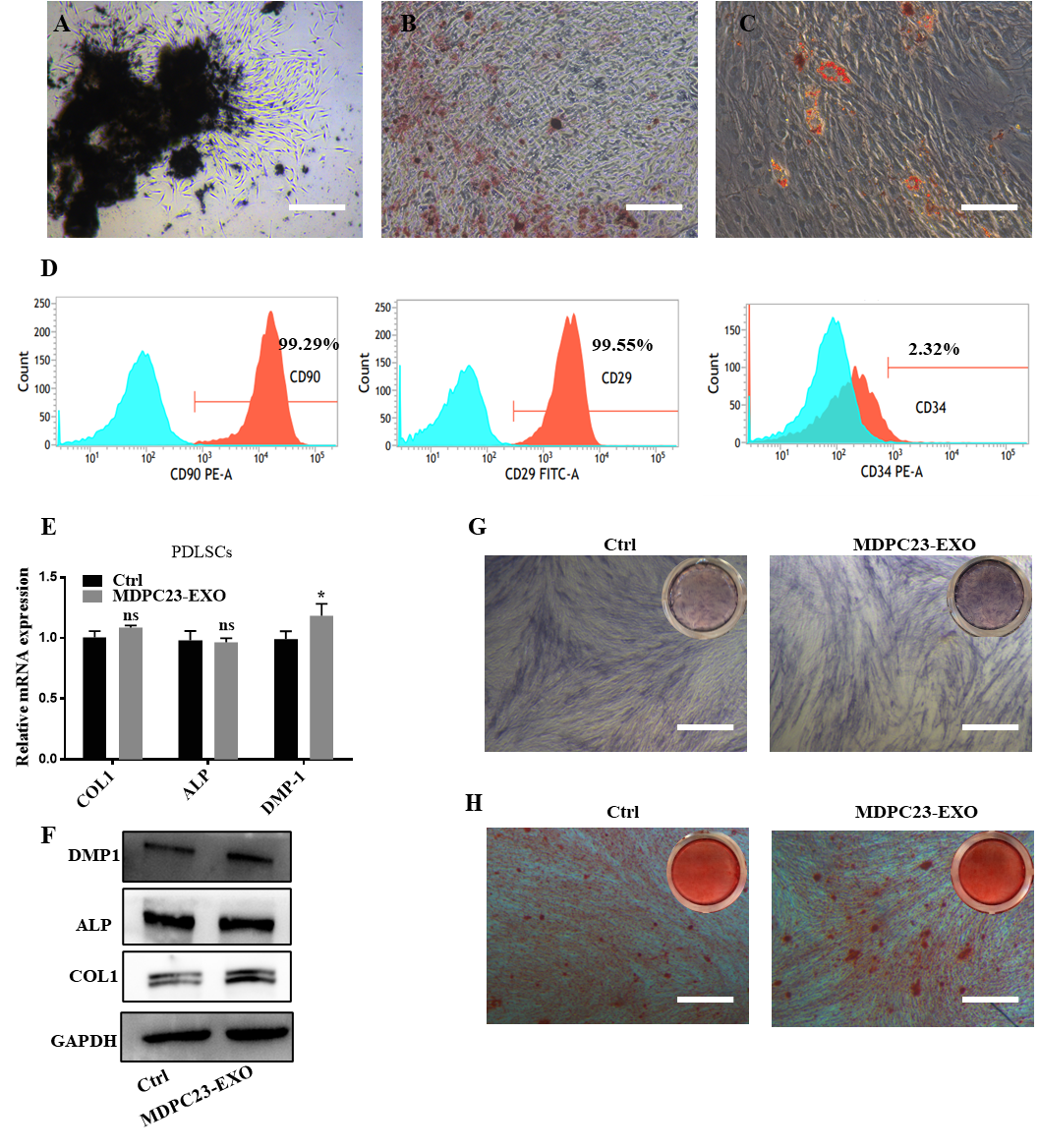
**

**Supplementary Figure 7.** PDLSCs were identified and the mineralization ability of PDLSCs was not inhibited by exosomes that were derived from MDPC23 cells. (A): Primary cultured PDLSCs (scale bar = 400 *μ*m). (B): Alizarin Red S staining showed that the induced PDLSCs could form mineralized nodules (scale bar = 400 *μ*m). (C) The adipogenic differentiation of the cultured PDLSCs was assessed by Oil red O staining, and the results showed the formation of clustered lipid droplets (scale bar = 200 *μ*m). (D) Surface marker expression of PDLSCs were measured by flow cytometric analysis. PDLSCs were positive for CD29 and CD90 expression and negative for CD34 expression. (E) PDLSCs were cocultured with 10^8^ particles/mL exosomes in mineralized-induced medium. qRT‒PCR analysis showed that the mRNA expression level of ALP was not inhibited by exosomes that were derived from MDPC23 cells and that the mRNA expression levels of COL1, ALP and DMP1 were promoted by exosomes that were derived from MDPC23 cells. (F) The western blotting results showed that the expression levels of ALP, COL1 and DMP1 were not inhibited by exosomes that were derived from MDPC23 cells. (G, H) ALP staining and Alizarin Red S staining showed that the ALP activity and mineralized nodule formation of PDLSCs were not inhibited by exosomes that were derived from MDPC23 cells (scale bar = 400 *μ*m).
